# The concept of personal drugs in the undergraduate pharmacology practical curriculum

**DOI:** 10.4103/0253-7613.45157

**Published:** 2008

**Authors:** D.M. Parmar, S.P. Jadav

**Affiliations:** Department of Pharmacology, M.P. Shah Medical College, Jamnagar-361 008, Gujarat, India. E-mail: drdinesh06@rediffmail.com

This is with reference to the correspondence, ‘The concept of personal drugs in the undergraduate pharmacology practical curriculum’.[1] We would like to highlight the following facts:

The usual dose recommendation of tinidazole in acute amoebic dysentery is 2 gm/day for 3 days.[2] The author has mentioned it as 1 gm/day for 3 days. It is obvious that the average cost per course with tinidazole (Rs. 21-25) would obviously be doubled in acute amoebic dysentery, i.e. Rs. 42-50, as compared to the average cost per course with metronidazole (Rs. 6.75-27.00).

The author has not searched the prices of the mentioned formulations meticulously. The price of two tablets of 1 gm tinidazole is Rs. 8.74.[3] Therefore, the average cost per course with tinidazole for acute amoebic dysentery would be Rs. 26.22. Similarly, the author has mentioned Rs. 35.00 for 2 gm of secnidazole, while it is available at Rs. 26.40.

Metronidazole is given in doses of 400 to 800 mg, three times daily, orally for five to 10 days, in amoebiasis. An alternative to this regimen is 1.5 to 2.5 gm as single daily dose for two or three days.[4] A dose of 2.4 gm metronidazole in a single daily dose for three days is equally effective to a standard recommendation of 5-7 days' therapy in acute intestinal amoebiasis.[5] This indicates that metronidazole 2.4 gm, once daily for three days, is quite cost effective as compared to tinidazole 2 gm once daily for three days (Rs. 11.20 versus Rs. 26.22). Amoebic liver abscess has been treated successfully by short courses (2.4 gm once daily for two days) of metronidazole or tinidazole.[6]

The standard treatment for invasive amoebiasis is metronidazle, ornidazole or tinidazole, followed by a luminal amoebicide, to eradicate any surviving organisms from the lumen of the large intestine and prevent relapse.[4] Treatment with tissue amoebicide should always be followed by a course of a luminal amoebicide, to eradicate the source of the infection.[7]

To choose a P-drug for a specified condition is one of the steps of the process of rational treatment. In our previous article, we have included the process of choosing a P-drug for acute amoebic dysentery and not a whole process of rational treatment of that condition. Therefore, the author's comment on this part may not be appropriate. Detailed information is found in Chapter 1 (The process of rational treatment) and Chapter 3 (Example of selecting a P-drug for angina pectoris) of ‘Guide to good prescribing; A practical manual’.[8]
